# Efficacy and limitations of systemic corticosteroids in patients with CRSwNP compared to alternative therapies with monoclonal antibodies: real-life of 101 patients from the Lazio region, Italy

**DOI:** 10.3389/falgy.2025.1573764

**Published:** 2025-06-16

**Authors:** F. Anastasi, C. Di Nota, S. Sessi, A. Marzetti, A. Sambito, M. Della Casa, S. Pizzolante, G. Bandiera

**Affiliations:** ^1^ Unit of Otorhinolaryngology, Medical and Surgical Rhinology, Ospedale San Giovanni Evangelista, Tivoli, Italy; ^2^ Department Faculty of Medicine, Unicamillus—Saint Camillus International University of Health and Medical Sciences, Rome, Italy; ^3^ Unit of Otorhinolaryngology and Maxillofacial Surgery, Ospedale F. Spaziani- Frosinone, Frosinone, Italy; ^4^ Unit of Otorhinolaryngology, Ospedale Belcolle ASL Viterbo, Viterbo, Italy; ^5^ Otolaryngology Unit, Department of Neuroscience, Mental Health and Sensory Organs (NESMOS), Faculty of Medicine and Psychology, Sapienza University, Rome, Italy

**Keywords:** CRSwNP, Th2 inflammation, systemic corticosteroid, pharmaco economic, biological therapy, asthma, IL-5 antibody, anti-IL-4

## Abstract

Chronic Rhinosinusitis with Nasal Polyps (CRSwNP) is a chronic inflammatory disease. Its severe uncontrolled form may share, with asthma and atopic dermatitis, the pathophysiological mechanism of T helper 2 inflammation, characterized by tissue eosinophilia and high local IgE levels. Comorbidities and the severity of symptoms result in a poor quality of life. CRSwNP is difficult to treat both medically and surgically. Systemic corticosteroids are widely used to manage signs and symptoms of this disease. However, prolonged use of systemic corticosteroids is associated with numerous side effects that limit their long-term use. Alternative therapy with new biologic drugs allows good control of the signs and symptoms of CRSwNP, without the side effects of systemic corticosteroids. Through an interview proposed to patients affected by CRSwNP, the use of systemic corticosteroids, their dosage and the methods of administration were analyzed in the period preceding the start of therapy with biological drugs and the real impact of biological therapy on corticosteroid use.

## 1 Introduction

Chronic rhinosinusitis with nasal polyps (CRSwNP) is a chronic inflammatory disease of the nose and paranasal sinuses characterized by symptoms (nasal obstruction, rhinorrhea, facial pain) for a period ≥3 months (1).

The prevalence of CRSwNP in the general population varies from 5%–15%, indicating its widespread presence in the world population. Nasal obstruction, continuous secretion, and the lack of smell present in the patient affected by CRSwNP determine a poor quality of life (HRQoL) (2). In addition to the physical symptoms, particular attention should be paid to the psychological and social discomfort that derives from it. In fact, these symptoms not only affect respiratory function, but also interfere with fundamental daily activities such as sleep, work and social interactions. The psychological impact of the disease is equally significant: depression, anxiety and frustration are common in patients with CRSwNP, fueling a vicious cycle of physical and psychological discomfort. Quality of life measures, through the Sino-Nasal Outcome Test (SNOT-22) and other validated tools, permit quantification of the effect of the disease. The CRSwNP guidelines recommend different treatment depending on disease severity: initially nasal irrigation with saline solution and topical intranasal corticosteroids (INCS).

For severe uncontrolled forms, short courses of oral corticosteroids are used (3, 4). Such cycles can rapidly reduce inflammation and improve the quality of life of patients. Patients refractory to medical therapy may benefit from endoscopic sinus surgery (ESS), although relapse rates are approximately 40% of patients within 18 months of ESS and nearly 80% within 12 years (5, 6). The number of repeat surgeries is higher among patients with type 2 inflammation, a condition exacerbated by intolerance to nonsteroidal anti-inflammatory drugs, comorbid disease (N-ERD), increased eosinophil counts, or higher levels of interleukin IL-5 and immunoglobulin IgE in nasal or sinus tissue (5, 7, 8). Common adverse events (AEs) associated with prolonged use of systemic corticosteroids have been well documented and, together with the associated costs, highlight the need for corticosteroid-sparing therapies (9, 10). Systemic corticosteroids are effective in reducing the size of the nasal polyp and improving symptoms, and therefore remain the first-line conservative treatment option for CRSwNP. Several studies have shown a positive impact, following this therapy, on the specific and general quality of life (SNQOL, GQOL) and on the nasal polyp score (NPS) in patients with CRS (11–13). The literature also shows that the combination of systemic and topical steroids may be superior to topical steroids alone (14) but this effect often does not last and many patients are at risk of developing side effects that significantly impact (15) on the quality of life, in some respects even more than other chronic diseases such as chronic obstructive pulmonary disease (COPD) (16).

The pathogenesis of chronic rhinosinusitis with nasal polyps (CRSwNP) is characterized by complex immune dysregulation and airway epithelial barrier disruption, driven by type 2 inflammation. The presence of eosinophils and the role of T cell subsets, particularly an imbalance between Treg and Th17 cells, are crucial to the pathogenesis of the disease. Eosinophils play a critical role in triggering type 2 inflammation. The immune response involves Th2 cells, eosinophils and IgE, among others, all activated by genetic and environmental factors. The intricate interplay between these elements, cytokines and innate lymphoid cells causes airway inflammation and hyperresponsiveness, contributing to the pathogenesis of eosinophilic asthma (17). Type 2 cytokines, such as interleukin-4 (IL-4), interleukin-5 (IL-5), and interleukin-13 (IL-13), play various functions within the inflammatory process (18), attracting eosinophils into tissues, resulting in the distinctive clinical symptoms observed in chronic inflammatory airway diseases.

Corticosteroids work by completely inhibiting the inflammatory cascade, reducing the infiltration of inflammatory cells into the nasal and sinus tissues. In addition, systemic corticosteroids are effective in reducing the size of nasal polyps and improving nasal airway patency, allowing patients to breathe better and reducing the need for surgery (19, 20).

Long-term use of systemic corticosteroids in patients with CRSwNP is associated with numerous potentially serious side effects, which may impair quality of life and increase morbidity. Main side effects include iatrogenic Cushing's syndrome, with increased body weight, skin fragility, and changes in fat distribution (21), endocrine and metabolic disorders with a predisposition to hyperglycemia and diabetes mellitus, as well as an increase in blood pressure.

Systemic corticosteroids weaken the immune response, increasing the risk of bacterial and fungal infections, particularly in the upper respiratory tract.

Recent studies have shown that, although effective in the treatment of CRS/WNP, chronic use of systemic corticosteroids must be carefully monitored. An analysis by Matsuwaki et al. emphasized the importance of limiting the duration of corticosteroid use to less than 6 weeks to reduce the risk of significant side effects (22), while the study by Liu et al. (23) suggested that low-dose intermittent corticosteroids may be equally effective, but with a better safety profile.

Intranasal corticosteroids offer local control of inflammation with significantly less risk of systemic side effects (24–28).

## 2 Materials and methods

### 2.1 Study design

This unaffiliated study used a cross-sectional survey design to understand how systemic corticosteroid use has changed in Italian patients with CRSwNP who are currently undergoing biological therapy. The study began in February 2024 and ended in June 2024.

The survey instrument was designed using Google Forms. The final draft of the survey underwent rigorous review by the authors, who are ear, nose, and throat (ENT) specialists. These specialists are actively involved in the management of patients with CRSwNP in hospital centers in the Lazio region of Italy, thus bringing a concrete estimate of real-life regional practice to the survey on this topic.

The patient recruitment phase took place from February 1, 2024–June 30, 2024, followed by consistent collection of results over the survey distribution period. During this period, patients were interviewed directly by their physicians during routine follow-up check-ups or contacted via email or WhatsApp, providing them with a direct link to the Google Forms survey. This multiple approach facilitated the data collection process, considered comprehensive and flexible, adapting to the different preferences and needs of patients.

### 2.2 Statistical analysis

For this study, only descriptive statistics were used (using Google Forms) and no minimum sample size was required.

## 3 Results

### 3.1 Diagnosis, symptoms and impact on quality of life

**Figure 1:**
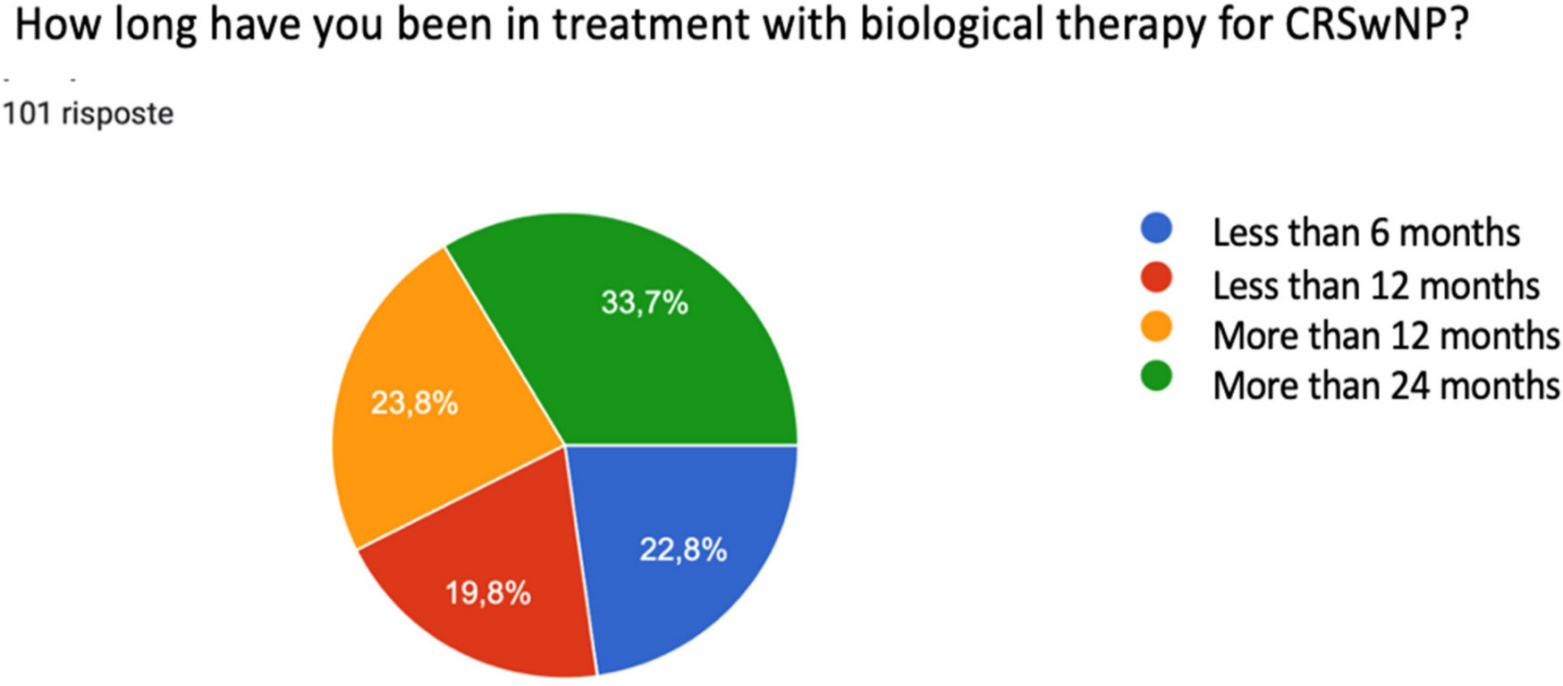
Impact of biological therapy in patients with CRSwNP.

The survey, completed and returned by 101 respondents, revealed that the majority of recruited patients, affected by CRSwNP (NPS > 5; 100% coexisting asthma) had been undergoing biological therapy for more than 24 months (33.7%), in 23.8% for more than 12 months. Patients who were pregnant or breastfeeding, younger than 18 years of age, treated with another biological drug in the current or previous 6 months for the treatment of Th2 inflammation, immunosuppressive treatment, any therapy for the treatment of oncological pathology in the current or previous 12 months, or autoimmune pathologies were not included in the study because they were not eligible for biological therapy for CRSwNP. The group of patients on biological therapy for less than 12 months was constituted by 19.8% patients who had recently started therapy with monoclonal antibodies for less than 6 months were also selected, which affected the total enrolled population at 22.8% (Figure 1).

## 4 Therapy with corticosteroids

Treatment with systemic corticosteroids (SCS) had been received by 101/101 participants for the control of CRSwNP, before starting treatment with biological therapy. Of these, 30% reported having taken Betamethasone, 24.3% were treated with Deflazacort, and 2.9% were prescribed Triamcinolone. 42.8% of participants reported having taken different SCS molecules over the years, prescribed by their doctor, without specifying which ones.

### 4.1 Symptoms alleviated by corticosteroid therapy

**Figure 2:**
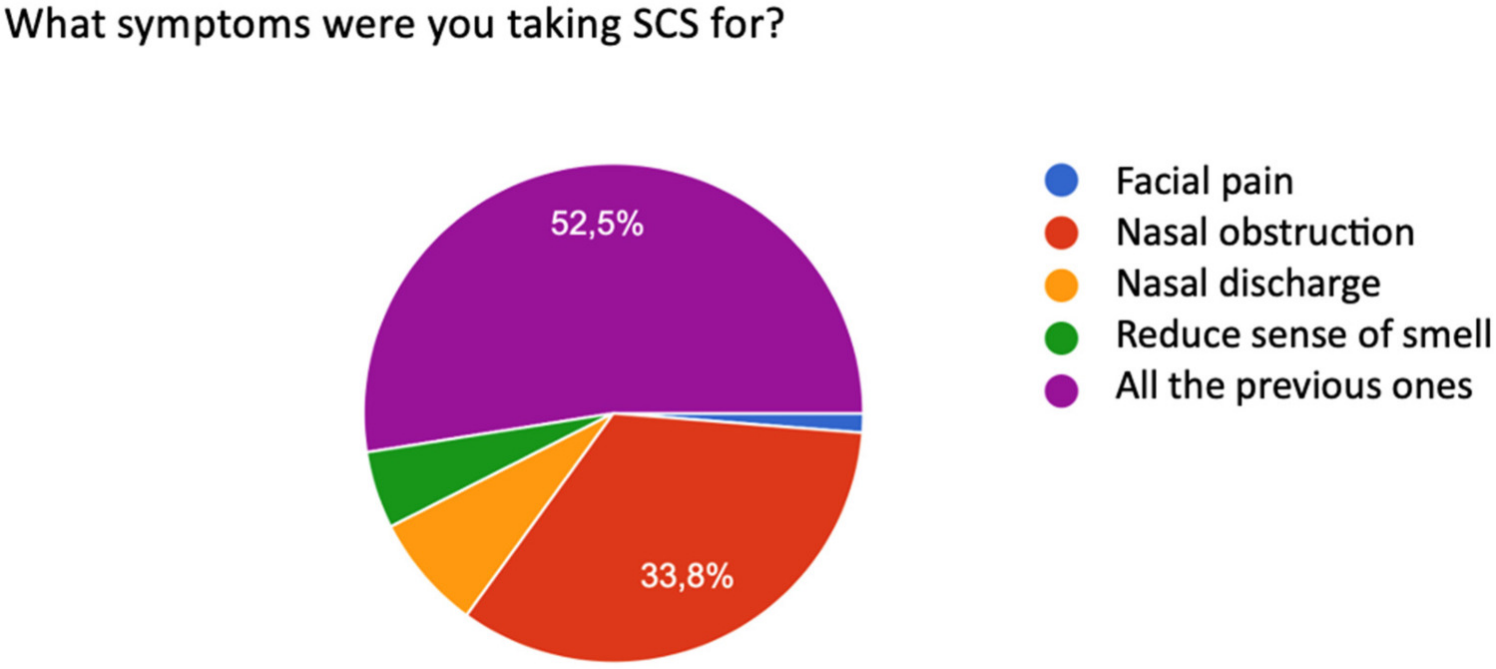
Distribution of symptoms reported by patients receiving corticosteroid therapy (SCS).

When asked for which symptom SCS was needed, patients responded as follows: 52.5% to relieve multiple symptoms simultaneously present in the exacerbation phase: nasal obstruction, facial pain, nasal secretions and perceived hypo-anosmia. 47.5% of patients reported the prevalence of only one symptom that determined the use of SCS therapy, nasal obstruction being the prevalent symptom, reported by 33.8%. 13.7% reported the prevalence of nasal discharge (anterior and/or posterior), followed by reduced sense of smell (11) and facial pain (Figure 2).

### 4.2 Effect of corticosteroids on symptom reduction

**Figure 3:**
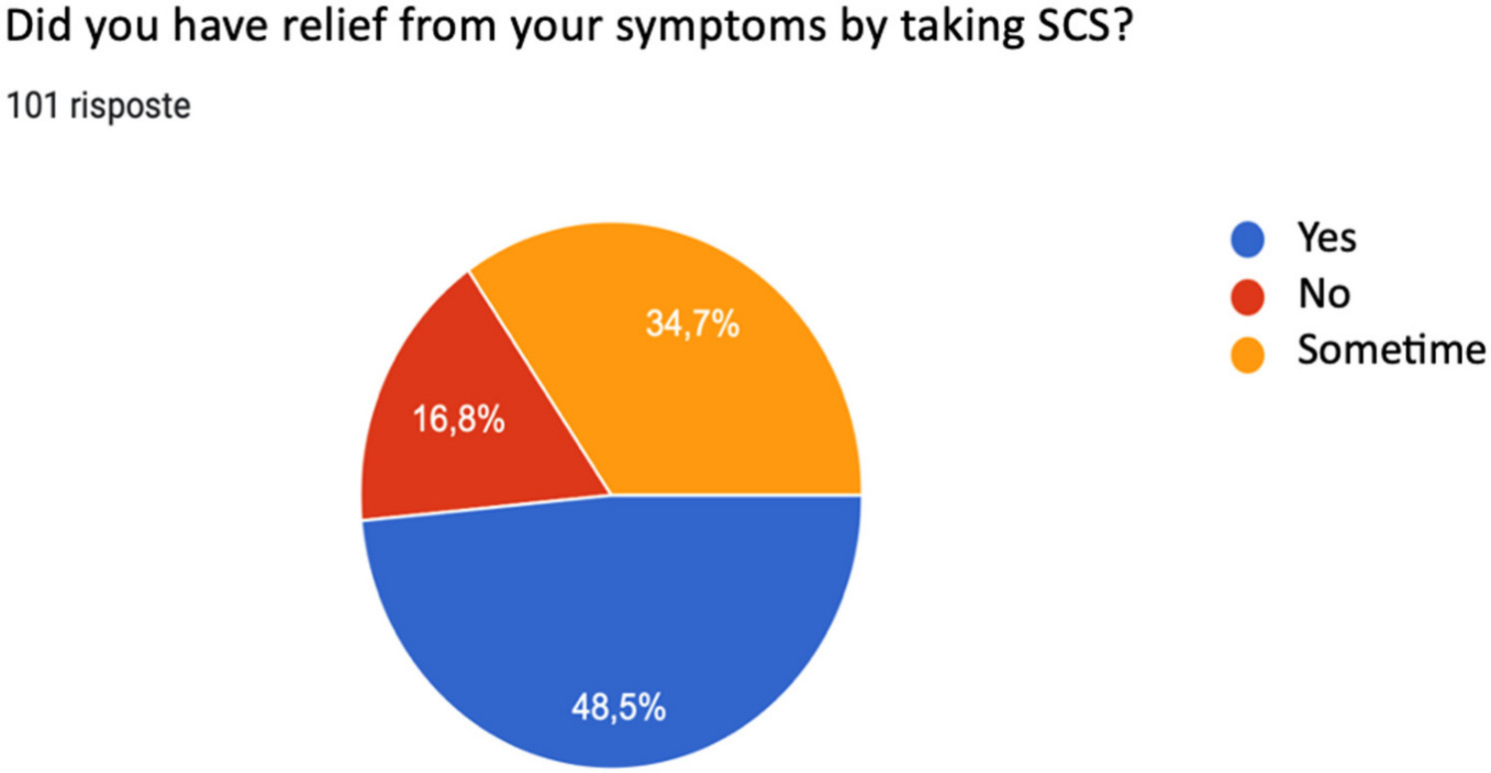
Effect of systemic corticosteroid (SCS) therapy on symptom reduction.

When asked whether taking systemic corticosteroids alleviated symptoms related to CRSwNP, 48.5% responded that they had actually benefited from the therapy, 34.7% reported that SCS therapy did not always lead to remission of symptoms with consequent objective relief, and finally 16.8% reported no benefit from corticosteroid therapy (Figure 3).

### 4.3 Dosage schedules

**Figure 4:**
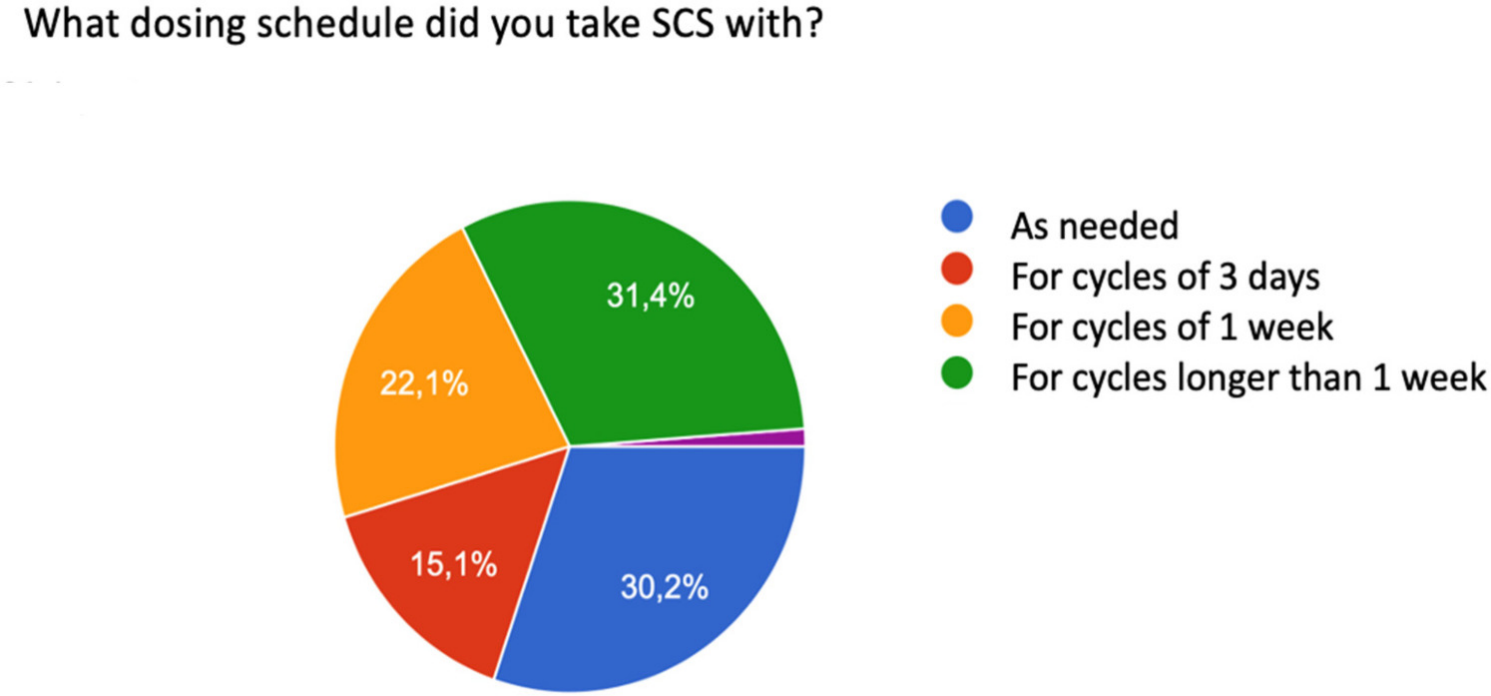
Patient-reported SCS dosing schedules.

26.8% of participants reported taking more than one cycle during the year, while 35.6% of patients had taken the drug more than once every 6 months and 37.6% with an almost monthly frequency. In addition, they had reported different dosage schedules in taking systemic corticosteroids: 31.4% for cycles longer than 7 days, 30% took the systemic corticosteroids as needed, 22.1% for cycles of one week, and 15.1% for cycles of three days. 1, 2% does not specify dosage regimen (purple slice) (Figure 4).

**Figure 5:**
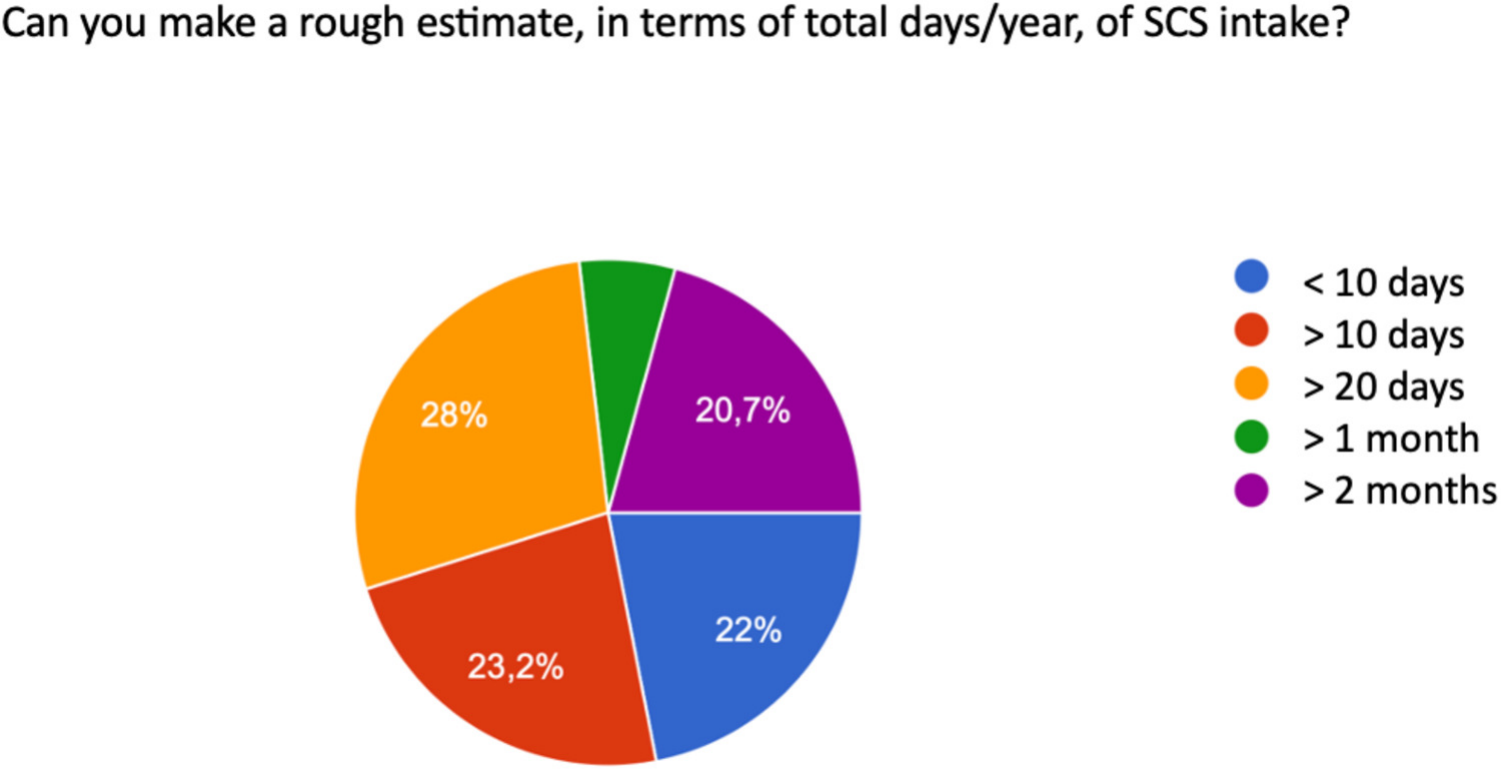
Total days/year of SCS use.

These values are summarized in an approximate estimate that considers the total days of systemic corticosteroid use, compared to the calendar year, in the following way: 22% therapy less than 10 days per year, 23.2% greater than 10 days per year, 28% greater than 20 days per year, and 20.7% use for a period greater than two months per year (Figure 5).

### 4.4 Method of corticosteroid therapy prescription

**Figure 6:**
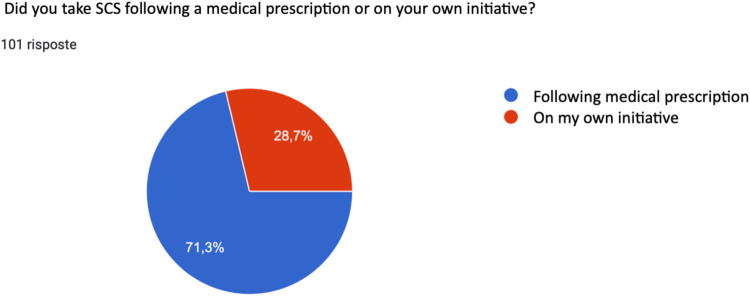
Method of prescription of SCS therapy.

When asked whether they had taken the systemic corticosteroid following a medical prescription, 71.3% of patients confirmed this, while 28.7% of those interviewed declared that they had taken the systemic corticosteroid on their own initiative (Figure 6).

### 4.5 Impact of biological therapy on corticosteroid use

When asked whether the patient had reduced systemic corticosteroid use since the start of biological therapy for the treatment of CRSwNP, the distribution of responses was as follows: 77.2% of participants stated that they had no longer taken systemic corticosteroids, 3% responded that the drug intake had been reduced by 50% in terms of duration of therapy, 2% reported the reduction reduced to 50% in terms of dosage of systemic corticosteroids, 17.8% responded by stating that they no longer took systemic therapy, but used the corticosteroids only as a topical spray to support biological therapy, as included in the current therapeutic plan.

## 5 Surgery

In the interview proposed to the participants, the surgical history for CRSwNP was not assessed, because it was not the object of this study.

## 6 Biological therapy

**Figure 7:**
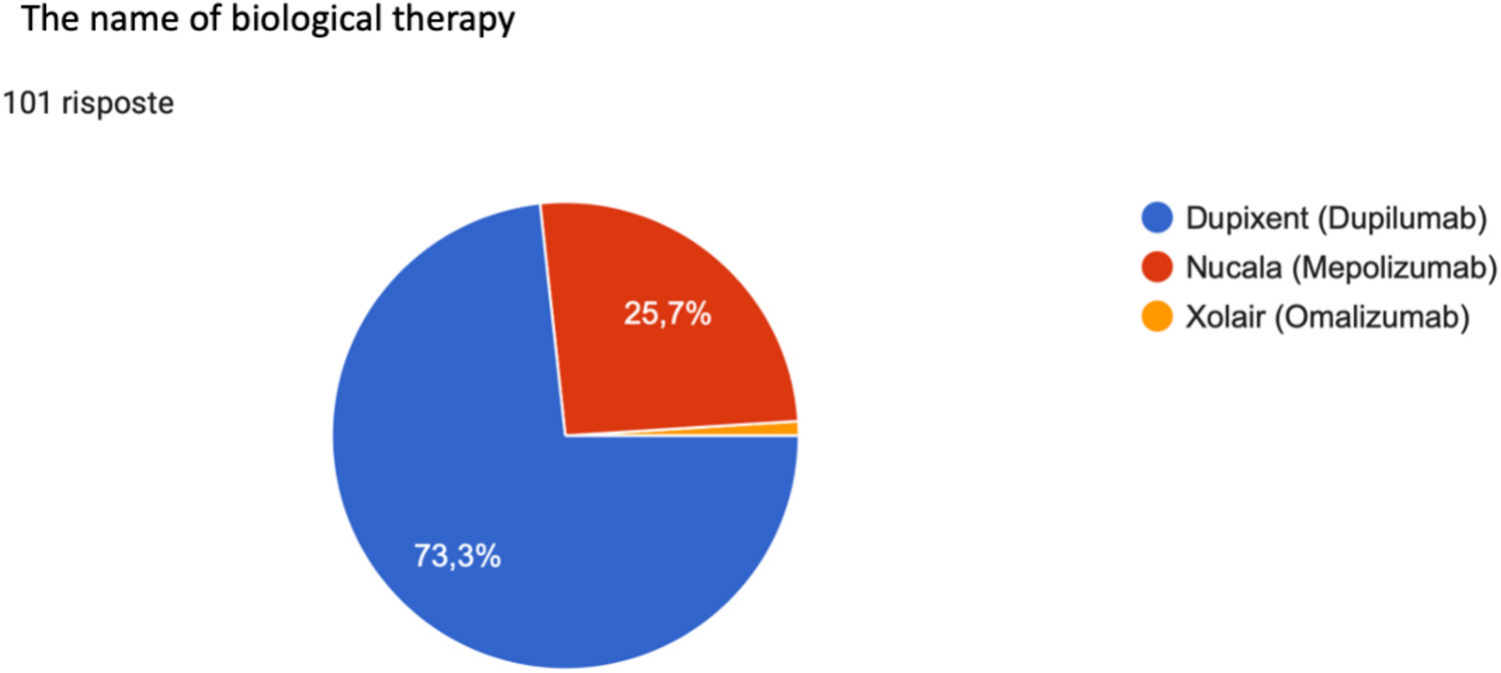
Distribution of biological drugs in the sample of patients affected by CRSwNP, attending the involved centers.

Regarding biological treatment at the centers involved in the study, 73.3% of patients were treated with Dupilumab (Dupixent), 25.7% were treated with Mepolizumab (Nucala), and 1% of patients were treated with Omalizumab (Xolair) (Figure 7).

## 7 Discussion

Our study, involving 101 patients with CRSwNP living in the Lazio region, Italy, provides valuable information on the administration of systemic corticosteroid therapy and the impact of biological therapy. Chronic Rhinosinusitis with Nasal Polyps (CRSwNP) is a chronic inflammatory condition involving the nasal cavity and paranasal sinuses, with the formation of nasal polyps. It is one of the most severe forms of chronic sinusitis and is associated with debilitating symptoms such as nasal obstruction, loss of smell, nasal discharge, and facial pain. The impact of CRSwNP is not limited to physical symptoms but can significantly impair patients' quality of life (QoL).

Systemic corticosteroids are the gold standard for the management of severe CRSwNP, as they can rapidly reduce inflammation and improve patients' quality of life. However, long-term use of these drugs is complicated by systemic side effects and, in addition, the benefit obtained by the patient is lost when therapy is suspended. Therefore, when the form of CRSwNP falls within a type 2 inflammation, in order to meet the need for long-term outcomes, even greater than the benefit that can be obtained from surgical treatment, alternative treatments for CRSwNP have been developed. These include monoclonal antibodies, taken by the patient in association with intranasal corticosteroid: this therapeutic approach allows systemic and local control of inflammation with a significantly lower risk of systemic side effects (27). It is worth remembering that in the most severe cases, endoscopic surgery for the removal of nasal polyps remains an effective solution for disease control (24).

From a cost analysis, systemic corticosteroids such as prednisone, frequently prescribed during CRSwNP exacerbations, must be taken for repeated courses of therapy due to the chronic nature of the disease, which leads to a significant increase in drug purchase costs. A recent review showed that, in the long term, pharmacological management of CRSwNP can lead to high costs per patient, obviously depending on the frequency and duration of treatments.

In addition to the primary direct cost of medication, follow-up visits and additional treatments to monitor corticosteroid side effects, such as osteoporosis, hypertension, and metabolic disorders, also add to the cost burden. Indirect costs associated with managing CRSwNP are also significant. Reduced work productivity, sick days, and absences from work are a significant economic burden, in addition to the serious side effects of corticosteroids, which can lead to a deterioration in quality of life, resulting in increased use of healthcare resources (11, 29, 30).

The use of intranasal corticosteroids or biologics such as anti-IL-5 drugs (e.g., mepolizumab) or anti-IL-4/IL-13 (dupilumab) have led to a reduction in the intake of systemic corticosteroids (14), thus reducing long-term costs and minimizing systemic side effects.

Our study originated from the intent to understand how well the signs and symptoms of CRSwNP were controlled by systemic corticosteroid therapy and how much biological therapy had a positive impact on disease control, all in a patient-subjective perspective. The data collected allow us to confirm that patients affected by CRSwNP require corticosteroids that are taken differently in terms of dosage and duration (31). The benefits reported by patients are related to the use of SCS but disappear with the suspension of the therapy. This leads the patient to start a new cycle of cortisone. The lack of an alternative medical therapy has also led to an inconsiderate use of corticosteroids over the years and the easy supply of this drug has frequently allowed autonomous management, without considering the consultation with the referring physician essential. This conduct has led, in some cases, to the development of other systemic pathologies typical of prolonged and/or inappropriate use of corticosteroids. It is probable that the lack of medical supervision in the use of the therapy did not allow the patient to recognize the side effects that arose. For these reasons, patients have sometimes continued to take systemic corticosteroids for the treatment of CRSwNP, not considering the side-effect pathologies developed as a result of poorly controlled use of SCS. The arrival of alternative therapies, such as monoclonal antibodies, has represented a new opportunity to treat the signs and symptoms of CRSwNP, without developing the known side effects of systemic corticosteroid intake.

From a cost perspective, by ensuring good control of the disease and associated comfort without the side effects of SCS, biological therapy allows long-term economic savings both in direct costs and in indirect costs resulting from type 2 inflammatory diseases. In terms of quality of life, the psychophysical well-being that biological therapy has brought to patients affected by CRSwNP is periodically confirmed by the score of tests such as Snot 22. There is evidence of a saving induced by biological therapy even if the estimate is calculated in Qualy (32).

Systemic corticosteroids act on nasal obstruction, olfactory dysfunction, facial pain, nasal secretions. The manifestation of these symptoms can be very different from patient to patient, and often there is a prevalence of one symptom over the others. Of our interviewees, 52.5% were using medical therapy for the resolution of all symptoms related to CRSwNP, considered equally debilitating, while 33.8% considered nasal obstruction the predominant symptom overall. Although corticosteroid therapy is the medical gold standard for the treatment of severe CRSwNP, in a percentage of patients it does not lead to symptom reduction and, if the pathology is eosinophilic, the recurrence of symptoms after suspension of therapy is more frequent (11, 33). Our survey presented data consistent with findings by Head and colleagues who noted that three to six months after treatment with oral steroids, patients showed little or no improvement in health-related quality of life (HRQoL) or symptom severity compared with those treated with placebo or no treatment (34). We consider that 48.5% of our interviewees reported that they had not obtained any benefit from systemic corticosteroid therapy.

The data that particularly struck us was the frequency of steroid intake. If Epos/Euforea 2023, among the criteria for prescribing biological therapy, consider the intake of systemic corticosteroid in the last year for a period equal to or greater than three months, 26.8% of the participants in our survey reported having had more than 1 cycle of therapy during the year, 35.6% of patients took the drug more than once every 6 months, 37.6% used steroid therapy with almost monthly frequency.

These data are consistent with the extreme variability of systemic corticosteroid dosage highlighted in the article by De Corso et al (31). Our interview showed that 30.2% of patients took the drug as needed. This data also suggested that the patient can take a drug with significant side effects independently, regardless of a medical prescription, as stated in the next question. The appropriateness of prescribing the subsequent biological therapy is confirmed by 100% of the patients interviewed who no longer had to resort to systemic corticosteroids to relieve symptoms related to the rhino-sinus pathology, after starting therapy with the monoclonal antibody. These patients, as per the therapeutic plan, were prescribed topical intranasal corticosteroids.

The guidelines indicate that the lack of efficacy of medical therapy with systemic corticosteroids indicates surgical treatment. When in the long term, surgery does not lead to lasting results (35) and when surgical revision has been necessary, biological therapy has shown promising results in managing not only the signs of symptoms related to chronic rhinosinusitis with nasal polyposis but also in controlling asthma and atopic dermatitis, comorbidities of type 2 inflammation. Biological treatments have demonstrated lasting improvements in symptoms and clinical signs for these patients (36, 37).

Having found no benefit from medical treatment with systemic corticosteroid or surgical treatment, our interviewees started therapy with: Dupilumab, anti-IL-5 antibody (73.3%) (56), with Mepolizumab, anti-IL-4R antibody (25.7%), and Omalizumab anti-IgE antibody in a minimal percentage.

Current guidelines for prescribing and monitoring biological therapy in CRSwNP are based primarily on scientific literature, with some differences due to different interpretations of criteria and definitions of treatment thresholds. Roland et al. proposed a therapeutic algorithm that includes assessment and management of comorbidities, medical management, and initiation of biologic therapy (40). Recommendations for the management of CRSwNP are provided by EPOS 2020, updated in 2023 (41). It suggests using biologic therapy for patients with CRSwNP who meet at least three of the following five criteria: (1) type 2 inflammation; (2) need for oral glucocorticoids; (3) significant impact on quality of life (QoL); (4) severe loss of smell; (5) diagnosis of concomitant asthma. Additionally, the Joint Task Force on Practice Parameters provides guidelines on the use of intranasal corticosteroids, biologics, and aspirin therapy after desensitization (ATAD) for the management of CRSwNP (42).

The lack of standardization in international guidelines and treatment protocols for CRSwNP may lead to variable therapeutic pathways from one country to another.

These gaps highlight the urgent need to develop more effective and personalized therapeutic strategies for patients with CRSwNP, inspired by models already adopted for other chronic diseases such as asthma, diabetes, chronic kidney disease, Parkinson's disease and coronary heart disease. These diseases require personalized diagnostic and care pathways, including regular screening (38, 39), symptom monitoring, management of comorbidities, patient education and psychological support. The therapeutic plan in use in Italy takes into account these unmet needs, indicating the prescribability of biological drugs to patients who have not had benefits from treatment with systemic corticosteroids and/or surgical treatment; patients who have a poor quality of life, as attested by tests such as Snot 22, which also takes into account the psychological aspects and limitations that chronic rhinosinusitis with nasal polyposis can determine.

## 8 Conclusions

This survey-based study of individuals living with CRSwNP provides valuable information on the impact of systemic corticosteroid therapy in this patient population and the extent to which biologic therapy impacted their quality of life, considering olfactory dysfunction, nasal obstruction, nasal discharge, and signs and symptoms related to comorbidities.

Treatment of CRSwNP often requires multiple and potentially indefinite therapies.

Systemic corticosteroids are an effective treatment for severe CRSwNP but their prolonged use is associated with numerous side effect, in the range of 10%–70%, depending on the duration and dosage of therapy. This is why short cycles are generally recommended for acute symptom control. It is critical to balance the therapeutic benefits with the risks and try to minimize the use of systemic steroids through therapeutic alternatives, such as intranasal corticosteroids, surgery, and monoclonal antibodies (53–55). While systemic corticosteroids are effective in the short-term control, monoclonal antibodies offer a safer option for the long-term treatment of CRSwNP, with a significantly lower incidence of severe side effects. Taking biologic therapy in patients with the severe, non-responsive forms of standard of care guarantees significant outcomes. These molecules are generally well tolerated and have a better safety profile, with generally mild and transient side effects. Incidence rates vary depending on the drug and the population studied, in the range of 0.1%–10% (43–51).

Clinicians should carefully monitor patients on treatment and consider the use of more targeted treatments to avoid long-term complications.

Management of CRSwNP with systemic corticosteroids entails significant direct and indirect healthcare costs, which are influenced by the long-term side effects of such treatments. The adoption of alternative treatments and strategies for early monitoring and management of side effects could reduce the overall economic burden (52). The continuous search for more effective and safe therapeutic solutions is essential to improve the quality of life of patients and contain the healthcare costs associated with this chronic disease.

## Data Availability

The original contributions presented in the study are included in the article/Supplementary Material, further inquiries can be directed to the corresponding author.

## References

[1] Desrosiers M Mannent LP Amin N Canonica GW Hellings PW Gevaert P Dupilumab reduces systemic corticosteroid use and sinonasal surgery rate in CRSwNP. Rhinology. (2021) 59(3):301–11. 10.4193/Rhin20.415 33847325

[2] Wang E-T Zheng Y Liu P-F Guo L-J. Rinosinusite cronica eosinofila negli asiatici orientali. World J Clin Cases: WJCC. (2014) 2:873. 10.12998/wjcc.v2.i12.873 25516863 PMC4266836

[3] Fokkens WJ Lund VJ Bachert C Mullol J Bjermer L Bousquet J EUFOREA consensus on biologics for CRSwNP with or without asthma. Allergy. (2019) 74(12):2312–9. 10.1111/all.13875 31090937 PMC6972984

[4] Orlandi RR Kingdom TT Hwang PH Smith TL Alt JA Baroody FM International consensus statement on allergy and rhinology: rhinosinusitis. Int Forum Allergy Rhinol. (2016) 6(S1):S22–S209. 10.1002/alr.21695 26889651

[5] Calus L Van Bruaene N Bosteels C Van Zele T Holtappels G De Vos G Twelve-year follow-up study after endoscopic sinus surgery in patients with chronic rhinosinusitis with nasal polyposis. Clin Transl Allergy. (2019) 9:30. 10.1186/s13601-019-0268-5 31249662 PMC6570859

[6] Hopkins C Slack R Lund V Brown P Copley L Browne J. Long-term outcomes from the English national comparative audit of surgery for nasal polyposis and chronic rhinosinusitis. Laryngoscope. (2009) 119(12):2459–65. 10.1002/lary.20653 19780032

[7] Bachert C Zhang N Hellings PW Bousquet J. Endotype-driven care pathways in patients with chronic rhinosinusitis. J Allergy Clin Immunol. (2018) 141(5):1543–51. 10.1016/j.jaci.2018.03.004 29731100

[8] Tokunaga T Sakashita M Haruna T Asaka D Takeno S Ikeda H Novel scoring system and algorithm for classifying chronic rhinosinusitis: the JESREC study. Allergy. (2015) 70(8):995–1003. 10.1111/all.12644 25945591 PMC5032997

[9] Canonica GW Colombo GL Bruno GM Di Bona D Di Matteo G Blasi F Shadow cost of oral corticosteroids-related adverse events: a pharmacoeconomic evaluation applied to real-life data from the severe asthma network in Italy (SANI) registry. World Allergy Organization Journal. (2019) 12(1):100007. 10.1016/j.waojou.2018.12.001 30937132 PMC6439414

[10] Manson SC Brown RE Cerulli A Vidaurre CF. The cumulative burden of oral corticosteroid side effects and the economic implications of steroid use. Respir Med. (2009) 103(7):975–94. 10.1016/j.rmed.2009.01.003 19372037

[11] Fokkens WJ Lund VJ Hopkins C Hellings PW Kern R Reitsma S European position paper on rhinosinusitis and nasal polyps 2020. Rhinology. (2020) 58(Suppl S29):1–464. 10.4193/Rhin20.600 32077450

[12] Chong LY Head K Hopkins C Philpott C Burton MJ Schilder AG Different types of intranasal steroids for chronic rhinosinusitis. Cochrane Database Syst Rev. (2016) 4:CD011993. 10.1002/14651858.CD011993.pub2 27115215 PMC8939045

[13] Vaidyanathan S Barnes M Williamson P Hopkinson P Donnan P Lipworth B. Treatment of chronic rhinosinusitis with nasal polyposis with oral steroids followed by topical steroids: a randomized trial. Ann Intern Med. (2011) 154:293–302. 10.7326/0003-4819-154-5-201103010-00003 21357906

[14] Gurnell M Radwan A Bachert C Lugogo N Cho SH Nash S Dupilumab reduces asthma disease burden and recurrent SCS use in patients with CRSwNP and coexisting asthma. J Asthma Allergy. (2024) 17:1–8. 10.2147/JAA.S420140 38250137 PMC10799571

[15] Mueller SK Wendler O Mayr S Traxdorf M Hosemann W Olze H Effect of postoperative systemic prednisolone on short-term and long-term outcomes in chronic rhinosinusitis with nasal polyps: a multi-centered randomized clinical trial. Front Immunol. (2023) 14:1075066. 10.3389/fimmu.2023.1075066 36969262 PMC10032209

[16] AlBloushi S Al-Ahmad M. Exploring the immunopathology of type 2 inflammatory airway diseases. Front Immunol. (2024) 15:1285598. 10.3389/fimmu.2024.1285598 38680486 PMC11045947

[17] Maspero J Adir Y Al-Ahmad M Celis-Preciado CA Colodenco FD Giavina-Bianchi P Infiammamma di tipo 2 nell’asma E in altre malattie delle vie aeree. ERJ Open Res. (2022) 8(3):00576–2021. 10.1183/23120541.00576-2021 35923421 PMC9339769

[18] Guo CL Liu FF Wang DY Liu Z. Type 2 biomarkers for the indication and response to biologics in CRSwNP. Curr Allergy Asthma Rep. (2023) 23(12):703–13. 10.1007/s11882-023-01114-w 37987873

[19] Minagawa S Araya J Watanabe N Fujimoto S Watanabe J Hara H Real-life effectiveness of dupilumab in patients with mild to moderate bronchial asthma comorbid with CRSwNP. BMC Pulm Med. (2022) 22(1):258. 10.1186/s12890-022-02046-3 35764984 PMC9241284

[20] Zhou L Zhang X Wang Y Chen D Liu Z Bachert C Osteoporotic fractures and corticosteroid use in chronic rhinosinusitis patients: a longitudinal study. Osteoporos Int. (2023) 34(7):1327–35. 10.1007/s00198-023-06731-9

[21] Nakashima D Nakayama T Minagawa S Adachi T Mitsuyama C Shida Y Dupilumab improves eosinophilic otitis media associated with eosinophilic chronic rhinosinusitis. Allergol Int. (2023) 72(4):557–63. 10.1016/j.alit.2023.03.007 37061391

[22] Campion NJ Brugger J Tu A Stanek V Brkic FF Bartosik TJ The "real life" efficacy of dupilumab is independent of initial polyp size and concomitant steroids in CRSwNP. J Otolaryngol Head Neck Surg. (2023) 52(1):56. 10.1186/s40463-023-00663-4 37674253 PMC10481502

[23] Liu Y Wang X Zhang L Bachert C Gevaert P Han P The efficacy and safety of systemic corticosteroids in chronic rhinosinusitis with nasal polyps: a systematic review and meta-analysis. J Allergy Clin Immunol. (2023) 151(2):548–56. 10.1016/j.jaci.2022.10.023

[24] Kato A Peters AT Stevens WW Schleimer RP Tan BK Kern RC Mechanisms of nasal polyp formation in chronic rhinosinusitis with nasal polyps: therapeutic implications. Allergy. (2023) 78(5):1056–68. 10.1111/all.15678

[25] Matsuwaki Y Ookushi T Asaka D Yoshimura T Kikuchi S Moriyama H Short-term corticosteroid treatment in chronic rhinosinusitis with nasal polyps: efficacy and safety. Int Forum Allergy Rhinol. (2022) 12(4):352–8. 10.1002/alr.22912

[26] Rondón C Campo P Eguiluz-Gracia I Plaza C Bogas G Torres MJ Long-term effects of systemic corticosteroids in CRS with nasal polyps: a retrospective cohort study. Eur Respir J. (2021) 58(3):190–7. 10.1183/13993003.01902-2020

[27] Singh SK Smith TL Rank MA Kita H Carter RG Stevens WW Systemic corticosteroid-induced immunosuppression and risk of infections in chronic rhinosinusitis patients. Clin Immunol. (2023) 156(2):123–30. 10.1016/j.clim.2023.109876

[28] Anastasi F Canevari FRM Gallo S Gramellini G Heffler E La Mantia I Olfactory impairment in Italian patients with chronic rhinosinusitis with nasal polyps: a patient-centered survey. Front Allergy. (2025) 5:1519069. 10.3389/falgy.2024.1519069 39840273 PMC11747549

[29] Gokcan A Mace JC Smith TL Alt JA Ramakrishnan VR Soler ZM The economic burden of chronic rhinosinusitis with nasal polyps in the United States. Int Forum Allergy Rhinol. (2019) 9(8):860–5. 10.1002/alr.22345

[30] Suh JD Wu AW Taw MB Nguyen M Wang MB Patel ZM Economic impact of chronic rhinosinusitis and nasal polyps: a systematic review. J Allergy Clin Immunol. (2020) 145(2):358–66. 10.1016/j.jaci.2019.07.03231600545

[31] De Corso E Pipolo C Cantone E Ottaviano G Gallo S Canevari FRM Survey on use of local and systemic corticosteroids in the management of chronic rhinosinusitis with nasal polyps: identification of unmet clinical needs. J Pers Med. (2022) 12(6):897. 10.3390/jpm12060897 35743682 PMC9225345

[32] De Corso E Furneri G Salsi D Fanelli F Ronci G Sala G Cost-utility analysis of dupilumab for the treatment of chronic rhinosinusitis with nasal polyps (CRSwNP) in Italy. J Pers Med. (2022) 12(6):951. 10.3390/jpm12060951 35743736 PMC9225649

[33] Sun C Zhang L Zhang Y Wang Y Wang K Liu S Chronic rhinosinusitis with nasal polyps is associated with chronic otitis media in the elderly. Braz J Otorhinolaryngol. (2017) 83(1):66–72. 10.1016/j.bjorl.2016.01.01027166273

[34] Head K Chong LY Hopkins C Philpott C Burton MJ Schilder AG. Short-course oral steroids alone for chronic rhinosinusitis. Cochrane Database Syst Rev. (2016) 4(4):CD011991. 10.1002/14651858.CD011991.pub2 27113367 PMC8504433

[35] Rosenfeld RM Piccirillo JF Chandrasekhar SS Brook I Ashok Kumar K Kramper M Clinical practice guideline (update): adult sinusitis. Otolaryngol Head Neck Surg. (2015) 152(2_suppl):S1–S39. 10.1177/0194599815572097 25832968

[36] Bachert C Bhattacharyya N Desrosiers M Khan AH. Burden of disease in chronic rhinosinusitis with nasal polyps. J Asthma Allergy. (2021) 14:127–34. 10.2147/JAA.S290424 33603409 PMC7886239

[37] Wu Y Fu Y He Y Gong X Fan H Han Z Prevalence and influencing factors of sleep disorders in patients with CRS: a protocol for systematic review and meta-analysis. BMJ Open. (2023) 13(12):e078430. 10.1136/bmjopen-2023-078430 38159959 PMC10759058

[38] Van Regemorter V Hummel T Rosenzweig F Mouraux A Rombaux P Huart C. Mechanisms linking olfactory impairment and risk of mortality. Front Neurosci. (2020) 14:140. 10.3389/fnins.2020.00140 32153360 PMC7046549

[39] Choi JS Jang SS Kim J Hur K Ference E Wrobel B. Association between olfactory dysfunction and mortality in US adults. JAMA Otolaryngol Head Neck Surg. (2021) 147(1):49–55. 10.1001/jamaoto.2020.3502 33090196 PMC7582225

[40] Roland LT Pinto JM Naclerio RM. The treatment paradigm of chronic rhinosinusitis with nasal polyps in the COVD-19 era. J Allergy Clin Immunol Pract. (2020) 8(8):2492–4. 10.1016/j.jaip.2020.06.029 32592789 PMC7831784

[41] Hellings PW Lau S Scadding GK Bjermer L Backer V Chaker AM EUFOREA summit in Brussels 2023: inspiring the future of allergy & respiratory care. Front Allergy. (2023) 4:1236977. 10.3389/falgy.2023.1236977 37577332 PMC10415067

[42] Kim SL Rank MA Peters AT. The chronic rhinosinusitis practice parameter. Ann Allergy Asthma Immunol. (2023) 131(3):307–10. 10.1016/j.anai.2022.12.022 37667905

[43] Xie P Deng Y Liu J Yang X Zhang L Bachert C The impact of nasal polyps on quality of life in patients with chronic rhinosinusitis. Laryngosc Investig Otolaryngol. (2021) 6(5):1125–33. 10.1002/lio2.663

[44] Bachert C Han JK Desrosiers M Hellings PW Amin N Lee SE Biological therapies in chronic rhinosinusitis with nasal polyps. Curr Allergy Asthma Rep. (2021) 21(4):19. 10.1007/s11882-021-00997-x33666743

[45] Van Zele T Holtappels G Gevaert P Bachert C. Chronic rhinosinusitis with nasal polyps: quality of life and its predictors. Am J Rhinol Allergy. (2019) 33(6):680–8. 10.1177/1945892419863167 24980230

[46] Gevaert P Van Bruaene N Cattaert T Van Steen K Van Zele T Acke F Mepolizumab for chronic rhinosinusitis with nasal polyps (SYNAPSE): a randomized, double-blind, placebo-controlled trial. Lancet Respir Med. (2017) 5(6):471–80. 10.1016/S2213-2600(17)30174-128664847

[47] Fokkens WJ Han JK Wagenmann M Bachert C Hellings PW Lee SE Efficacy and safety of benralizumab in chronic rhinosinusitis with nasal polyps: the OSTRO randomized clinical trial. JAMA. (2020) 323(1):36–47. 10.1001/jama.2019.19356

[48] Bachert C Han JK Desrosiers M Hellings PW Amin N Lee SE Efficacy and safety of dupilumab in chronic rhinosinusitis with nasal polyps: results from the SINUS-24 and SINUS-52 trials. N Engl J Med. (2021) 384(3):202–13. 10.1056/NEJMoa2023694

[49] Gevaert P Calus L Van Zele T Bachert C Zhang N Mullol J Management of chronic rhinosinusitis with nasal polyps: current and future treatment options. J Allergy Clin Immunol. (2022) 150(1):96–106. 10.1016/j.jaci.2022.05.012

[50] Zhang N Van Bruaene N Bachert C Gevaert P Han JK Hellings P Biologics in the treatment of chronic rhinosinusitis with nasal polyps: a systematic review. Curr Opin Allergy Clin Immunol. (2021) 21(1):23–30. 10.1097/ACI.0000000000000705

[51] Fokkens WJ Lund VJ Mullol J Bachert C Alobid I Baroody F Chronic rhinosinusitis with nasal polyps: current concepts and management. Eur Respir J. (2020) 56(4):1900–8. 10.1183/13993003.01902-2020

[52] Galli J Ciofalo A Settimi S Di Cesare T Di Matteo G Paludetti G Economic burden of chronic rhinosinusitis with nasal polyps and associated comorbidities in Europe: a cost-of-illness analysis. Eur J Health Econ. (2023) 24(2):1–11. 10.1007/s10198-023-01573-y36346476

[53] Alangari AA, Alsaleh S, Wali S, Alroqi F, Almuhanna F, Alshamrani M. Targeting eosinophilic inflammation in chronic rhinosinusitis with nasal polyps: current and emerging therapies. J Allergy Clin Immunol. (2023) 152(3):665–74. 10.1016/j.jaci.2023.05.018

[54] Singh A Bansal S Chowdhury N Patel GB Laidlaw TM Peters AT Dupilumab in chronic rhinosinusitis with nasal polyps: a multicenter real-world evidence study. Annals of allergy. Asthma Immunol. (2023) 130(1):72–8. 10.1016/j.anai.2022.09.023

[55] Harris KA Maspero JF Bachert C Han JK Lee SE Sousa AR Tralokinumab in severe chronic rhinosinusitis with nasal polyps (TROPOS): a phase 3 randomized controlled trial. Lancet Respir Med. (2023) 11(5):364–72. 10.1016/S2213-2600(23)00084-7

[56] De Corso E Pasquini E Trimarchi M La Mantia I Pagella F Ottaviano G Dupilumab in the treatment of severe uncontrolled chronic rhinosinusitis with nasal polyps (CRSwNP): a multicentric observational phase IV real-life study (DUPIREAL). Allergy. (2023) 78(10):2669–83. 10.1111/all.15772 37203259

